# Mesophication in temperate Europe: A dendrochronological reconstruction of tree succession and fires in a mixed deciduous stand in Białowieża Forest

**DOI:** 10.1002/ece3.5966

**Published:** 2020-01-10

**Authors:** Andreea P. Spînu, Mats Niklasson, Ewa Zin

**Affiliations:** ^1^ Southern Swedish Forest Research Centre Swedish University of Agricultural Sciences (SLU) Alnarp Sweden; ^2^ Chair of Silviculture University of Freiburg Freiburg Germany; ^3^ Nordens Ark Foundation Hunnebostrand Sweden; ^4^ Department of Natural Forests Forest Research Institute (IBL) Białowieża Poland

**Keywords:** age structure, disturbance regime, fire suppression, forest history, oak trees, pine trees

## Abstract

1. The shift from shade‐intolerant species to shade‐tolerant mesophytic species in deciduous and mixed forests of the temperate zone is well described in studies from North America. This process has been termed *mesophication* and it has been linked to changes in fire regime. Fire suppression results in the cessation of establishment of heliophytic, fire‐dependent tree species such as oak (*Quercus*) and pine (*Pinus*). Due to the scarcity of old‐growth forests in Europe, data on long‐term compositional changes in mixed forests are very limited, as is the number of studies exploring whether fire played a role in shaping the dynamics.
2. The aim of this study was to reconstruct tree succession in a 43‐ha natural mixed deciduous forest stand in Białowieża Forest (BF), Poland using dendrochronological methods. In addition, the presence of aboveground fire legacies (charred and fire‐scarred deadwood) enabled the fire history reconstruction.
3. Dendrochronological data revealed tree establishment (*Quercus*) back to the end of the 1500s and fires back to 1659. Under a regime of frequent fires until the end of the 18th century, only oak and pine regenerated, sporadically. A shift in the fire regime in the first half of the 19th century triggered oak and pine cohort regeneration, then gradually spruce (*Picea*) encroached. Under an increasingly dense canopy and less flammable conditions, regeneration of shade‐tolerant *Carpinus*, *Tilia,* and *Acer* began simultaneously with the cessation of oak and pine recruitment.
4. *Synthesis*. The study reports the first evidence of mesophication in temperate Europe and proves that fire was involved in shaping the long‐term dynamics of mixed deciduous forest ecosystems. Our data suggest that fire exclusion promoted a gradual recruitment of fire‐sensitive, shade‐tolerant species that inhibited the regeneration of oak and pine in BF.

## Graphical abstract

Our study reports the first evidence of mesophication in temperate Europe and proves that fire was involved in shaping the long‐term dynamics of a mixed deciduous forest ecosystem in that region. Using tree‐ring data, we show that subsequent to fire cessation, gradual encroachment of fire‐sensitive, shade‐tolerant tree species inhibited the regeneration of light‐demanding, fire‐adapted species such as oak and pine.

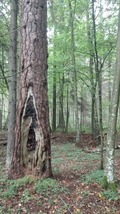

## 1 INTRODUCTION

Changes in forest composition were long‐discussed under unidirectional and cyclical ecological models based on the idea of a predictable forest development (Clements, 1916; Franklin et al., 2002; Kimmins, 1997), for example, phytosociological approaches based on forest typologies (Ellenberg, 1996). Such deterministic perspectives consider any event (disturbance) disrupting the “steady‐state” of the system as “exception” rather than as a factor acting over longer time periods and larger spatial scales (Oliver & Larson, 1996; White & Pickett, 1985). However, the above‐mentioned models assuming basically only one equilibrium point to which forest succession should lead have been challenged by an increasing number of ecological studies along with the theory of alternative stable states (Beisner, Haydon, & Cuddington, 2003). According to this theory, a community may exist in several possible configurations, that is, different equilibrium points that are locally stable and defined by a set of various variables (e.g., species abundance, age of populations, and spatial coverage) determined by biotic and abiotic factors. The community will stay in a certain state until a switch to another state will be triggered by a considerable change of conditions. Afterward either a return to the previous state or a development into a completely different configuration will occur. In the case of forest dynamics, disturbances (natural or man‐made) representing a substantial shift in conditions (White & Pickett, 1985) are now widely acknowledged as important drivers, shaping the succession both in time and space (e.g., Niklasson et al., 2010; Svoboda et al., 2013; Kuuluvainen, 2016). Variability in the characteristics of disturbance events and the resulting diversity of their legacies, as well as the existing species pool in the surrounding landscape, were recognized as crucial for shaping the following successional pathways of vegetation communities (e.g., Kuuluvainen, 2016; Pickett, Cadenasso, & Meiners, 2009). Among other disturbances affecting flora assemblages, fire—as one of the main plant biomass consumers—was proven to be of key importance in shaping vegetation patterns worldwide and to largely alter or even override both climatic and soil condition influences (Bond & Keeley, 2005; Nowacki & Abrams, 2015).

In temperate deciduous and mixed forests worldwide, great changes in the species composition have been noted—in North America (Varner et al., 2016), Europe (Brzeziecki, Pommerening, Miścicki, Drozdowski, & Żybura, 2016), and Asia (Bhatta & Vetaas, 2016). The general global pattern is a shift from shade‐intolerant species to more mesophytic species adapted to closed‐canopy (Brose, Dey, & Waldrop, 2014), described in the North American literature as the *mesophication* phenomenon (Nowacki & Abrams, 2008). In the current conditions of these forests, tree species such as oak (*Quercus*) and pine (*Pinus*) have ceased to regenerate and exclusively shade‐tolerant tree genera are successfully established (e.g., maple) (*Acer*) (Bobiec, Jaszcz, & Wojtunik, 2011; Brzeziecki et al., 2016; Kuijper, Cromsigt, et al., 2010; Nowacki & Abrams, 2008; Varner et al., 2016).

Although climate influence on mesophication cannot be excluded (Bhatta & Vetaas, 2016; Pederson et al., 2015), long‐term studies from temperate North America have linked this phenomenon mainly to changes in the disturbance regime (Flatley, Lafon, Grissino‐Mayer, & LaForest, 2015; Lafon, Naito, Grissino‐Mayer, Horn, & Waldrop, 2017; Nowacki & Abrams, 2008, 2015; Varner et al., 2016). Specifically, fire cessation imposed by law at the beginning of the 20th century has been proven to have a long‐term impact on forest composition and structure (McCullough, Werner, & Neumann, 1998). Fire affects the ecosystem's species composition on spatial and temporal scales (e.g., Lafon et al., 2017), being often a major cause of demographic bottlenecks for tree establishment by limiting the ability of woody vegetation to recruit from one demographic stage to the next—either by direct consumption, significant damage, or substantial modification of growth conditions (Bond & Keeley, 2005). The relationship between tree mortality and survival and, thus, canopy opening in the postdisturbance stand largely determines the abiotic conditions for regeneration (light, water, nutrients) (Keeley & Fotheringham, 2000; Pausas & Verdú, 2005) and the following tree regeneration patterns (sporadic or in cohorts) (Flatley, Lafon, Grissino‐Mayer, & LaForest, 2013; Storaunet, Rolstad, Toeneiet, & Blanck, 2013). Particularly, oak‐ and pine‐dominated forest stands have been shown to be strongly affected by changes in the fire regimes (Brose, Schuler, Van Lear, & Berst, 2001; Lageard, Thomas, & Chambers, 2000; Varner et al., 2016), as the colonization ability of those tree genera is strongly connected to fire by their life history and evolutionary traits (Agee, 1998; Brose et al., 2014; Fernandes, Vega, Jiménez, & Rigolot, 2008).

In temperate Europe, similar changes in tree species composition, from dominance of shade‐intolerant species to shade‐adapted species were revealed by long‐term inventories (approx. 80 years) of tree regeneration and mortality in primeval mixed stands of Białowieża Forest (BF) (Bernadzki, Bolibok, Brzeziecki, Zajączkowski, & Żybura, 1998; Brzeziecki et al., 2016). Given that the life span of most temperate tree species is within the order of centuries, shifts in species composition following shifts in disturbance regimes could be expected to operate over centuries or millennia rather than decades (Lafon et al., 2017). Detailed empirical data of forest and disturbance dynamics over longer time periods (centuries) are lacking from the deciduous habitats of BF. Such data exist from recent studies in coniferous stands, where a shift from fire‐adapted Scots pine (*Pinus sylvestris* L.) dominance to shade‐tolerant Norway spruce (*Picea abies* L. Karst.) have been shown to closely track changes in the fire regime (Niklasson et al., 2010; Zin, Drobyshev, Bernacki, & Niklasson, 2015; Zin, 2016). The ongoing compositional changes in the rich mixed deciduous forest component of BF has been widely discussed (Brzeziecki et al., 2016; Jaroszewicz, Bobiec, & Eycott, 2017), but not elucidated, especially regarding the long‐term perspective. Interestingly, and in contrast to conifer‐dominated landscapes in BF, this forest type consists of tree species with different life histories, that is, shade‐intolerant (*Quercus*, *Pinus*) and shade‐tolerant genera (*Acer*, *Tilia*, *Carpinus*), being fire‐adapted (pyrophytes) and fire‐sensitive taxa (mesophytes), respectively. Forest composition in those habitats was suggested to have been shaped either by browsing pressure (Bobiec, Kuijper, Niklasson, Romankiewicz, & Solecka, 2011b; Kuijper, Jędrzejewska, et al., 2010b), environmental factors, for example, climate or soil chemistry (Bernadzki et al., 1998), or changes in forest management (Brzeziecki et al., 2016). Fire has so far been neglected as a factor of importance in the studies of deciduous‐dominated stands (Bernadzki et al., 1998; Brzeziecki et al., 2016), despite numerous observations of fire legacies (scarred trees, stumps, and snags), which also occur in deciduous‐dominated portions of BF (Behrens, 2011; Faliński, 1986). Hence, past fire events might have played an important role in the dynamics of these mixed stands and changes in fire regime might have been involved in changes in the tree species composition similarly to temperate forests in North America.

The general objective of this study was to investigate the compositional changes of a mixed deciduous stand, consisting of species with contrasting life histories by reconstructing the long‐term tree population dynamics with dendrochronological data. The study also aimed to explore whether fire was involved in shaping the forest composition of this habitat type where signs of past fire in the form of fire‐scarred trees and stumps exist. To our knowledge, this is the first study investigating the role of fire in driving the long‐term changes of mixed deciduous forests of temperate Europe.

## 2 MATERIALS AND METHODS

### 2.1 Study area

**Figure 1:**
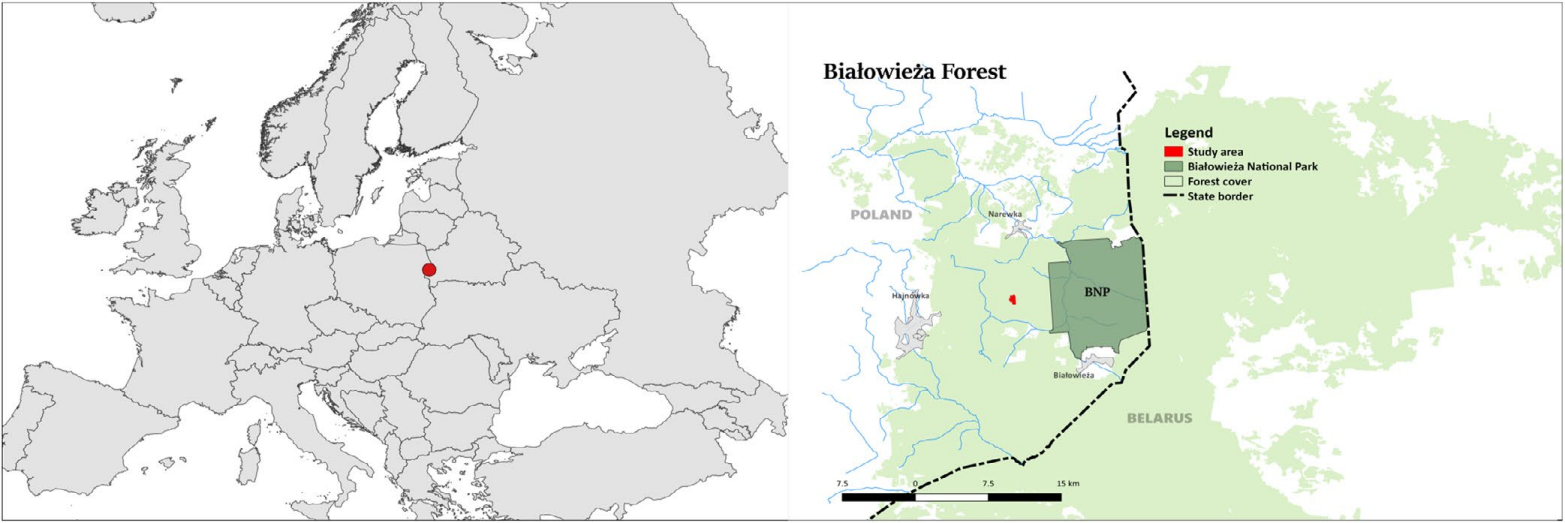
Location of Białowieża Forest (BF) in eastern Poland/western Belarus and location of the study area within BF

The rarity of natural mixed deciduous forests makes Białowieża Forest (BF) (52°30′‐53°N, 23°30′‐24°15′E, Poland/Belarus) (Figure 1) a valuable research area within temperate Europe. Thanks to its special protection status and well‐preserved stand structure (Faliński, 1986; Jędrzejewska & Jędrzejewski, 1998), BF bears a high potential for provisioning ecological reference data about the natural dynamics of such a forest type.

The climate of BF is transitional continental‐Atlantic, with a mean annual temperature of 7°C (−4.2°C in January and 17.7°C in July) (Faliński, 1986; Jędrzejewska, Jędrzejewski, Bunevich, Miłkowski, & Krasiński, 1997). The mean annual precipitation for the last five decades was 633 mm (Pierzgalski, Boczoń, & Tyszka, 2002) and the average annual snow cover period is 92 days (Faliński, 1986). The landform is rather uniform, as BF is situated on an old‐morainic plateau that originated from a glaciofluvial relief (135–190 m.a.s.l.) (Faliński, 1986). The soils range from podzols over mesotrophic and eutrophic brown and lessive soils to gley and organic soil types in the peatbog and semibog areas (Kwiatkowski, 1994).

Białowieża Forest's structural characteristics reflect a long‐term history of protection efforts and low‐intensity forest utilization. BF bypassed the intense colonization and implicit deforestation which occurred in nearly all European lowlands (Kaplan, Krumhardt, & Zimmermann, 2009). Anthropogenic changes occurred in the past, as the area was inhabited by people since early ancient times (Samojlik, 2006), but without modifying the intact character of the forest and its unique structure (Samojlik, Rotherham, & Jędrzejewska, 2013). Starting with the early 15th century, BF received a protection status under the Polish‐Lithuanian Commonwealth. Later, the Polish kings and the Russian tsars retained that status, mainly for exclusive royal hunting privileges (the European bison, *Bison bonasus* L. was and remains the flagship species of BF). Until the end of the 18th century, forest utilization was limited to small‐scale hay mowing, cattle pasturing, beekeeping and production of potash, tar, and charcoal. Although timber extraction was also performed in the area, patches of nonclear‐felled stands remained in what is today the core area of the Polish Białowieża National Park (BNP, established in 1921). Several surrounding stands were also left intact, many of which would become forest reserves at the end of the 20th and the beginning of the 21th centuries (Jaroszewicz, 2004; Samojlik, 2005). Such natural and seminatural old‐growth forest stands enclose valuable research sites for understanding long‐term forest dynamics through dendrochronological methods. Presence of fire‐scarred trees, stumps and snags have been noted in both coniferous and mixed deciduous stands of BF (Behrens, 2011; Faliński, 1986; Niklasson et al., 2010; Zin, 2016) enhancing the value of such sites for fire history reconstruction.

The study was conducted in a 43‐ha mixed deciduous forest stand with admixture of conifer trees and with well‐preserved old‐growth structures. The criteria for choosing the study site were the following: (a) low anthropogenic impact (uneven‐aged structure, presence of old, large tree individuals, deadwood diversity in amounts and quality: snags, stumps with potential long tree‐ring samples); (b) occurrence of direct fire evidence: fire‐scarred stumps and fire‐scarred trees.

According to the vegetation classification, the forest stand represents the *Tilio‐Carpinetum* type, a mesophilous deciduous forest community found on rich soils (Sokołowski, 1993). Ground vegetation is characterized by a dense herb layer, dominated by spring geophytes (*Anemone nemorosa* L., *Anemone ranunculoides* L., Isopyrum *thalictroides* L., *Gagea lutea* (L.) Ker Gawl., *Corydalis solida* (L.) Ker Clairv. and a continual layer of flora such as *Lamium galeobdolon* (L.) L., *Stellaria holostea* L., *Galium odoratum* (L.) Scop., *Hepatica nobilis* Mill., *Carex Pilosa* Scop., *Pulmonaria obscura* Dumort, *Asarum europaeum* L., *Oxalis acetosella* L., *Milium effusum* L., *Lathyrus vernus* (L.) Bernh. Despite the admixture of *Pinus* and *Picea*, there are no plans typical for coniferous forest communities (e.g., *Calluna vulgaris* (L.) Hull, *Vaccinium* spp., and feather mosses).

The stand density is approximately 580 N/ha, and the mean standing volume is 395 m^3^/ha. The basal area is dominated by pedunculate oaks (*Q. robur* L.). The uppermost tree canopy is dominated by old‐growth pedunculate oaks, Scots pine trees (*P. sylvestris* L.), and younger generation of Norway spruces (*P. abies* L. Karst). However, the pine population is not present throughout all the stand. Additionally, old, large individuals of small‐leaved lime (*T. cordata* Mill.) and Norway maple (*A. platanoides* L.) are scattered in the site. Admixture of silver birch (*Betula pendula* Roth) with high diameters is present in the canopy. The second tree layer consists of lime and hornbeam (*C. betulus* L.) and the understory is abundant with shrubs such as hazelnut (*Corylus avellana* L.), Spindle‐tree (*Euonymus* europaeus L. and *Euonymus verrucosus* Scop), daphne (*Daphne mezereum* L.), and dense regeneration of hornbeam and lime, especially in gaps. No young individuals of pine or oak with a height over 50 cm, which is lowest browsing‐height limit for red deer (*Cervus elaphus* L.) (Renaud, Verheyden‐Tixier, & Dumont, 2003), was spotted in the study site.

### 2.2 Fieldwork

#### 2.2.1 Long‐term tree population dynamics and fire history

Data describing the long‐term successional patterns were gathered from three circular 5,000 m^2^ sample plots (radius of 39.90 m). All trees with DBH (diameter at breast height, 130 cm above the ground) higher than 5 cm were inventoried. Up to a radius of 12.62 m (500 m^2^), all present tree species were cored with an increment borer as close to the mineral soil as possible (20–80 cm), whereas outside the inner 500 m^2^, only oak, pine, oak, and spruce individuals were cored. In total, we cored 275 trees at the ground level (one core per tree). To investigate the early growth of oak, pine, oak, and spruce on rich soils of BF and to determine the real ages, 42 trees were randomly selected from the trees cored at the ground level and they were cored additionally at DBH as well. Additionally, old‐growth trees of oak, pine, and birch were sampled subjectively inside the investigated area for a more robust representation of the long‐term stand history. Furthermore, to describe the current structure of the oak and pine populations as the potentially oldest tree generations in the study site (Faliński, 1986), a grid of parallel transects (N‐S direction) was established in the stand. The width of each transect was 50 m (25 m on each side of the walking line). Because pine trees were not present throughout all the study site, the transect length was delimitated by the pine presence (the inventory stopped at the last pine tree if no individuals were visible in the further 50 m). This threshold was convenient since pine trees are easy to distinguish among other trees due to their stem particularities and crown features. The area delimited by pine population was 27 ha. A precise inventory of all oak and pine trees (standing, living and dead trees and stumps) was carried out in the transects and their satellite positions were recorded. Any stem modifications suggesting past fire evidence were registered, including possible fire scars and charcoal presence (Kimmins, 1997). To explore the local fire history, all potentially fire‐scarred trees from the whole study area were cored. Cross sections from fire‐scarred pine stumps suitable for sampling and further analysis (*N* = 19) were subjectively collected with the chainsaw following the procedure of Arno and Sneck (1977) and McBride (1983).

#### 2.2.2 Tree regeneration at present

The complete inventory of pine seedlings and saplings was carried out during the tree inventory in transects. For the inventory of tree regeneration of other species on a macro‐scale, a transect of 25 m length and 2 m width was randomly established in the stand (orientated E‐W). All tree regeneration up to 5 cm stem diameter was recorded by species and height. The individuals (48) were collected for age estimation.

To get an indication of the early growth dynamics of the old pine and oak individuals on rich soils, the transect was examined for destructive sampling of seedlings, however no pine seedlings were found in the forest stand.

### 2.3 Laboratory work

#### 2.3.1 Dendrochronological analysis on increment cores and cross sections of trees

After discarding samples too degraded for analysis, 260 increment cores and 15 cross sections from Scots pine stumps were dried, glued on wooden mounts and sanded (down to 600 grit) for an easy reading of tree‐ring sequences. Under 6–40× magnification, the cores were visually cross‐dated by identifying local pointer years following the standard dendrochronological methodology of cross‐dating directly on the wood, without skeleton plots or tree‐ring width measurements (Stokes, 1996; Yamaguchi, 1991)—a method widely applied to date extreme events to which the trees were subjected, such as fires and/or to reconstruct forest succession (in Europe: Niklasson et al., 2010; Wallenius, Kuuluvainen, Heikkilä, & Lindholm, 2002; Zin et al., 2015). Examples of local pointer years, helpful for assigning calendar years to tree rings were: 1821/1822 [narrow sequence]; 1846/1847 [narrow sequence]; 1857 [narrow lw]/1858[narrow]; 1908 [narrow lw]/1909 [narrow]; 1928 [narrow lw or narrow]; 1940 [narrow in pine cores]; 1941 [narrow in spruce cores]; 1964 [narrow]; 1979 [narrow or missing]; 1996 [narrow]. Ages of trees at coring height were estimated according to the last year (pith). If the pith was missing, a pith locator was used to estimate the pith date (Applequist, 1958).

All developed fire scars found in increment cores and cross sections were noted (Niklasson & Granström, 2000). Additionally, the disturbances in tree‐ring morphology (Niklasson & Granström, 2000) or in the growth pattern were noted and used as a fire record if a certain year was confirmed by a fire scar formed in at least one other sample tree.

#### 2.3.2 Dendrochronological analysis on cross sections of seedling and saplings

The age of forty‐two hornbeam, one maple, and one lime seedlings, with heights between four and 52 cm was determined under high magnification. Samples were crosscut at root collar and the surface was sanded and smoothed with a scalpel cut to obtain the clearest possible view of the annual rings. Most samples were broadleaves (hornbeam and lime) with low contrast between cells and high occurrence of wedging, narrow and double rings. Thus, several laboratory procedures and methods were explored to obtain as high a ring visibility as possible. The most successful method involved the counting of rings under a water drop (Appendix S1). To determine the oldest pith date, the small‐sized samples were sectioned according to the root collar position (below, at and above root collar).

#### 2.3.3 Estimation of germination years

Germination years for oaks, pines, spruces, and hornbeams were calculated from the age at coring height corrected by local height growth models obtained from the sample of trees which were cored at two levels and from the aging of tree saplings (Appendix S2). Due to the lack of samples in the field and in the absence of prior empirical data on growth dynamics of different tree species in such rich sites with which to compare our data, the germination ages might have been under‐ or overestimated by 10 years. The species growth dynamics was very irregular. Spruce is a plastic species, which can show either a very suppressed or a very fast early growth (Niklasson, 2002; Silvertown & Charlesworth, 2009). However, the cores were extracted with high accuracy (most of the cores included pith or missed the pith by less than 30 mm) and the correction for the coring height was based on the estimation of the early growth rates of oak, pine and spruce on rich soils of BF, obtained from the trees cored at two levels. For hornbeam, we applied a simple linear regression model used to predict the age based on height, developed on the basis of the sampled seedlings and saplings.

To test the age difference, a nonparametric Kruskal–Wallis test was carried out since the data did not comply with the requirements for parametric test methods.

All data analyses were performed using SPSS Software (IBM Corp., 2016).

## 3 RESULTS

### 3.1 Successional patterns

**Figure 2:**
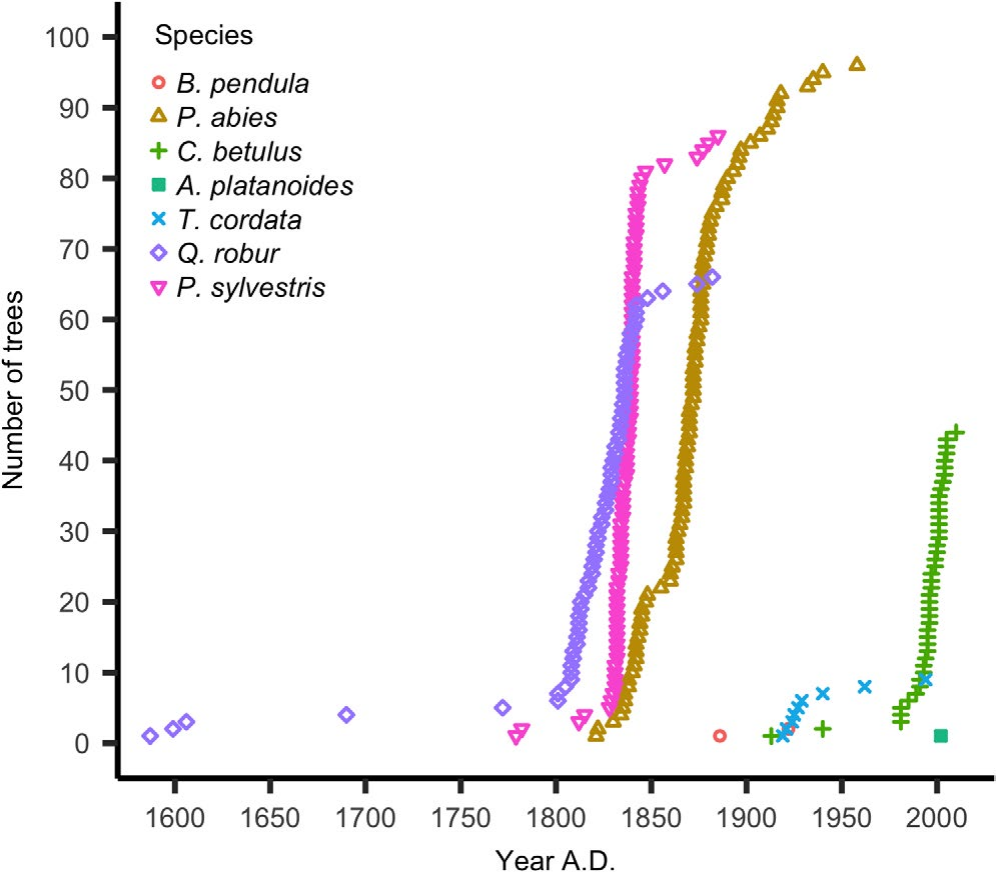
Cumulative tree germination derived from increment cores and sapling cross sections. *N* = 304

The presence of ancient trees and old deadwood allowed for reconstruction of more than 400 years of the stand's history and showed a long‐term shift from shade‐intolerant to shade‐adapted tree species. Sporadic regeneration of oak and pine occurred prior to the 1800s, followed by a rapid oak and pine regeneration with gradual encroachment of spruce during the 19th century (Figure 2). Oak and pine recruited in cohorts, starting in 1815 and 1830 for oak and in 1837 for pine. Approximately 80% of oaks and 85% of pines originated from the 1830s–40s. Generally, both oak and pine presented similar steep recruitment patterns, followed by a relaxation phase, which continued until the 1870s. The last regeneration of oak was in 1874 and of pine in 1888. Spruce regeneration started in the 1820s as a more gradual process. Regeneration waves of spruce continued to be observed in 1834, 1860 and in 1932. Along with the decline of oak and pine establishment in the 1870s–80s, sporadic spruce recruitment took place. Hornbeam and lime began to establish around the late 1920s, concurrent with a decrease in regeneration rates of spruce.

### 3.2 Current age structure

**Figure 3:**
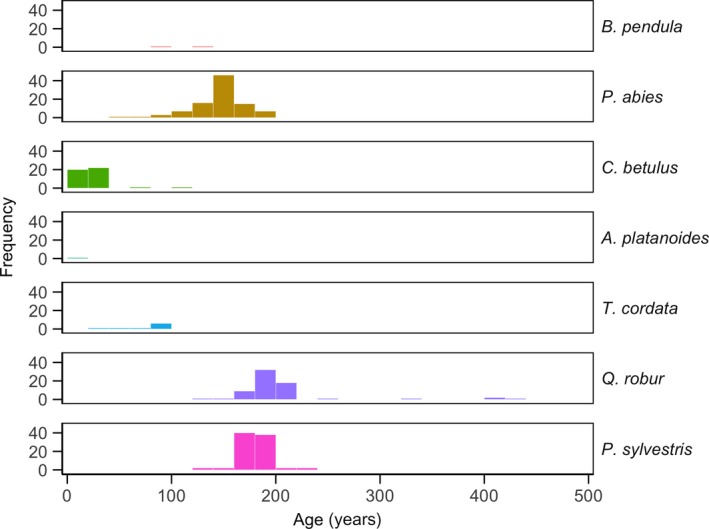
Age structure of the studied tree populations. *N* = 86 (*Pinus sylvestris*); *N* = 66 (*Quercus robur*); *N* = 9 (*Tilia cordata*); *N* = 44 (*Carpinus betulus*); *N* = 96 (*Picea abies*); *N* = 2 (*Betula pendula*); *N* = 1 (*Acer platanoides*)

**Figure 4:**
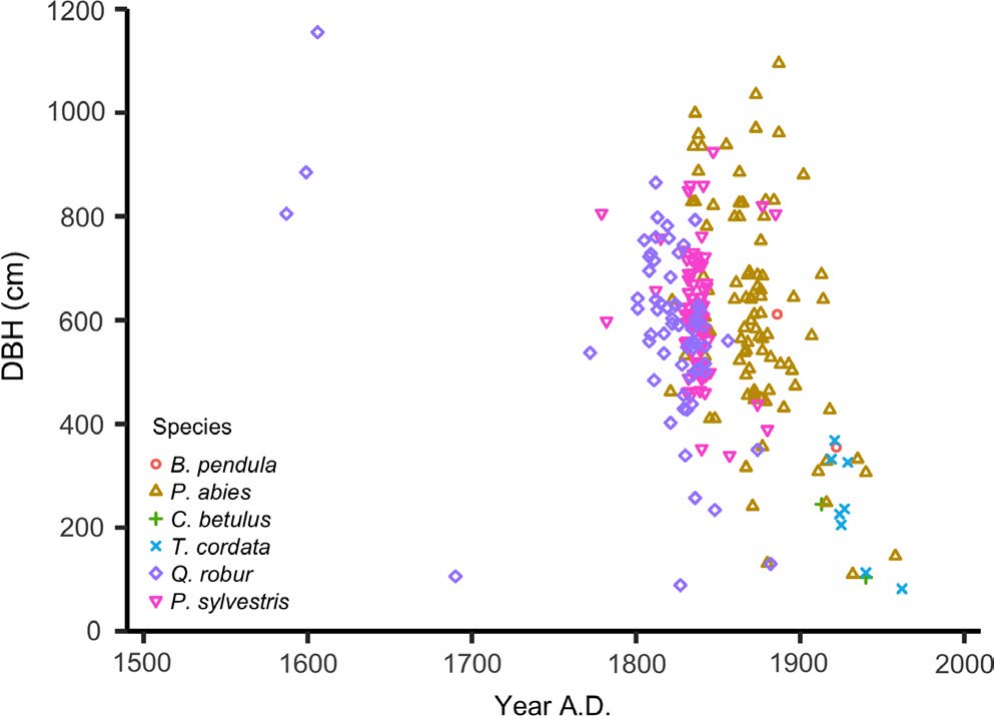
Diameter variation in relationship with germination year per species. *N* = 86 (*Pinus sylvestris*); *N* = 66 (*Quercus robur*); *N* = 8 (*Tilia cordata*); *N* = 2 (*Carpinus betulus*); *N* = 96 (*Picea abies*); *N* = 2 (*Betula pendula*)

The upper canopy layer was dominated by old cohorts of large‐diameter oaks and pines and a population of spruce with higher age and size variation. The lower layer was composed of younger hornbeam, lime and birch trees of lower diameters (Figures 3 and 4). The oldest trees in the canopy were oaks, whereas the spruces belonged to younger generations (Kruskal–Wallis, *df* = 2, *p* < .001*, *N* = 230). Most of the oak individuals belonged to age classes from 181 to 220 years and they displayed the features of a cohort regeneration. Thirty‐two oak individuals were dated to be over 200 years and three individuals over 400 years old. The current population of pine was characterized by trees with ages from 171 to 190 years, occurring in highest frequencies in the age class of 171–80 years. Age structure of spruce displayed a peak in the age class of 141–160 years and a variation from 60 to 190 years. Maximum values for tree ages were 430 years (oak), 238 years (pine) and 203 years (spruce). Neither oaks nor pines younger than 140 and 132 years respectively, were found. Other mature tree species sampled in the stand belonged to younger age classes: lime and hornbeam (61–100 years) and birch (two individuals only, of 95, respectively 131 years).

The current tree regeneration is highly dominated by hornbeam (share of more than 90%). The mean age of the saplings from the transect in the stand was 20 years (±1.02, min–max: 7–32 years) (data not shown). Other species found in the transect were lime and maple. Within the entire study area (43 ha), no oak or pine saplings (more than 50 cm in height) were found.

### 3.3 Fire evidence

**Figure 5:**
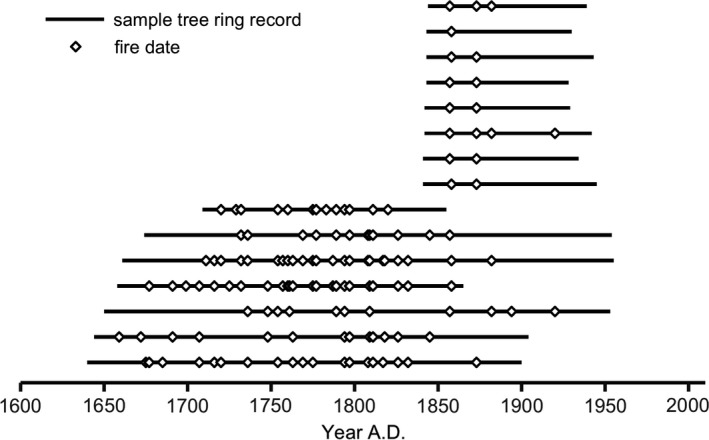
Fire history of the studied 43‐ha mixed deciduous forest stand in Białowieża Forest, NE Poland. Reconstructed by cross‐dating of pine stumps. *N* = 15

**Figure 6:**
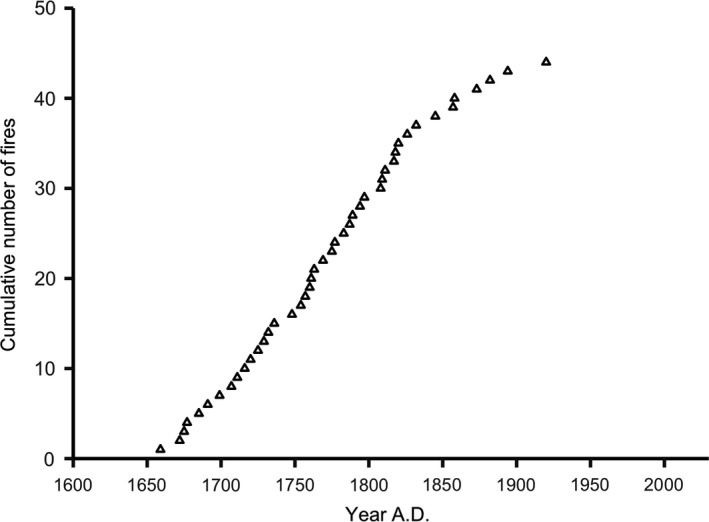
Cumulative number of fires over time recorded in a 43‐ha mixed deciduous forest stand in Białowieża Forest, NE Poland

From the 518 oak and pine stumps inventoried, 42 stumps of both species presented evidence of past fire occurrence in form of charcoal presence and/or possible fire scars (Appendix S3). Local fire history was reconstructed from 15 pine stumps preserved in a condition allowing for sampling and further analysis (Appendix S4). Fire scars and fire‐induced disturbances in the tree‐ring morphology were also found in the cores of pine, oak and spruce. In total, 44 different fire dates were recorded in the stand, with the earliest fire in 1659 and the latest one in 1920 (Figure 5). Fire disturbances occurred frequently over the period 1659–1920, every sixth year on average (mean fire interval: 6.1 years ± 4.87 *SD*). Fire intervals varied from 1 to 26 years. During 1659–1832 the stand scale fire interval averaged 4.8 years (±2.97 *SD*) and ranged from 1 to 13 years. Between 1832 and 1920, mean stand scale fire interval increased up to 12.6 years (±7.46 *SD*), ranging from 1 to 26 years. The minimum fire interval recorded (1 year) was observed both at stand and individual tree scale (stand scale: 1760/1761, 1808/1809, 1817/1818, 1857/1858; individual tree scale: 1760/1761, 1808/1809, 1817/1818). A shift in the fire regime occurred in the 1830s, with declining fire occurrence (i.e., increasing fire intervals) since then (Figure 6). At present, almost a century has passed since the last fire disturbance in the studied stand (which occurred in 1920).

## 4 DISCUSSION

The present study provides a high‐resolution reconstruction of over 400 years of dynamic tree succession showing a shift from dominance of pyrophytes (shade‐intolerant, fire‐adapted species) such as oak and pine to an increasingly higher share of mesophytes (shade‐tolerant, fire‐sensitive species) like lime, hornbeam, maple via a transitional phase of spruce codominance. The replacement of shade‐ intolerant long‐lived trees with shade‐tolerant mesophytes has large similarities with well‐described vegetation turnover in other temperate forest ecosystems (*mesophication*, Nowacki & Abrams, 2008). As will be discussed below we believe fire has been an important driver of the forest composition in the early part of our record, and the later loss of fire has been instrumental for the observed changes in species composition, which has not been described in Europe earlier. Our argumentation is built upon a solid record of fire events from the studied stand.

Tree succession was grouped into three main periods: (a) 1600–1830s with sporadic recruitment of oak and pine; (b) 1830s–1920 dominated by cohort regeneration of oak and pine with later spruce encroachment and (c) 1920s–to present with the gradual recession of oak, pine, and spruce in favor of hornbeam and lime. The highest number of fires occurred in the 18th and the 19th centuries (a, b), while in the latter period (c), we recorded fire events only at the beginning of the 20th century (1920 last fire record).

### 4.1 Oak and pine dominance up to the mid‐19th century

The oldest three living trees in the studied stand are oaks originating from the late 16th century, which puts them among the oldest published tree ages from BF (Keczyński, 2017). The oldest, large‐diameter oak individuals are dispersed over an area of 27 ha (data not shown) which illustrates well the sporadic and spatially sparse nature of oak regeneration prior to the 1800s. The presence of pine stumps irregularly scattered across the study site (data not shown) and originating from the same period indicates a similar sporadic and sparse recruitment pattern. Regeneration of both oak and pine benefits from canopy openness (Mason & Alía, 2000; Reyes & Casal, 2006; Varner et al., 2016), being prolific especially in larger gaps (Bobiec, 2007; Packham, Harding, & Hilton, 1992). The low capacity of these two species to establish in closed‐canopy deciduous forest has been proven in several studies (Brzeziecki et al., 2016; Kuijper, Cromsigt, et al., 2010). We suggest that under frequent burning, stand conditions were substantially different from the present and characterized by low tree density, high degree of canopy openness and grass‐dominated ground vegetation—as indicated for the coniferous habitats of BF (Zin, 2016; Zin et al., 2015). Noteworthy, Brincken (1826) noted the presence of pioneer tree species such as birch in deciduous stands of BF, again supporting the idea of a more open structure of those ecosystems. Based on the other existing evidence, like pollen and flora studies from BF which show higher proportion of *Gramineae* and other light‐demanding species indicating an open environment throughout the 18th–19th centuries (Błoński, Drymmer, & Ejsmond, 1888; Mitchell & Cole, 1998; Zin, 2016), we propose that the above‐mentioned picture of a fire‐shaped, park‐like oak‐pine stand may likely reflect a broader pattern holding for large sections of BF.

Our results reflect data reported from temperate forests of Eastern North America, where several species of oaks and pines established and maintained their populations under frequent fire disturbance over centuries (e.g., Hoss, Lafon, Grissino‐Mayer, Aldrich, & DeWeese, 2008.; Flatley et al., 2015; recently summarized by Lafon et al., 2017), profiting from open stand conditions shaped by surface fires (Nowacki & Abrams, 2008), while fire‐sensitive tree species which were likely to be regenerating as well could not survive due to the short fire intervals (Lafon et al., 2017 and references therein). This pattern, with fire acting as a filter in shaping species composition (McEwan et al., 2014), has been proven to be a landscape‐scale process, acting throughout a gradient of habitat types: from xeric to mesic sites (Flatley et al., 2015). Based on this we propose that the filtering role of fire in shaping the long‐term relation between fire‐adapted pine and fire‐sensitive spruce, documented in conifer habitats of BF (Niklasson et al., 2010; Zin, 2016; Zin et al., 2015), may well have acted exactly the same way in mesic, mixed deciduous and deciduous portions of the landscape and has assured long‐term oak and pine dominance over other tree taxa.

### 4.2 The transitional phase and the encroachment of spruce

Our on‐site record of fire history gives firm evidence that fires were not a point phenomenon but spread throughout the stand. However, the record gives little information on intensity and severity of the fires which can be important for overstory mortality as well as for the following regeneration patterns. The first abrupt change in the tree succession in our study site occurred in the first half of the 19th century with a rapid regeneration of both oak and pine, followed by spruce encroachment. Oak and pine cohorts appeared in the first decades of the 1800s after the recorded fires in 1811, 1826 and 1832, which may indicate that those disturbance events were of higher severity since oak and pine were shown to dominate the regeneration after high‐intensity fires (Wallenius et al., 2002; Ziobro et al., 2016). Still, longer fire‐free intervals do promote cohort regeneration as well (Aldrich, Lafon, Grissino‐Mayer, De Weese, & Hoss, 2010; Brown & Wu, 2005), so the observed pattern in the forest composition may reflect both the effect of a mixed‐intensity fire regime of BF (Zin et al., 2015) and the general relaxation in fire disturbance which started in the stand in the 1830s.

The sudden start of the oak cohort regeneration in the early 1800s is not however seen in the fire record as a change in fire frequency. Thus, we cannot out rule also other factors having impact on tree regeneration, including for example other plant consumers (i.e., browsing ungulates), as it has been proposed by earlier studies in BF (Churski, Bubnicki, Jędrzejewska, Kuijper, & Cromsigt, 2017; Kuijper, Jędrzejewska, et al., 2010b; see also the following discussion).

Besides herbivores, also clear‐cuts or other large‐scale disturbances like insect outbreaks and storms could have been described as driving the initiation of quick establishment of both oak and pine by creating suitable growth conditions for seedlings of these shade‐intolerant species. However, the first cohorts from our study area appeared shortly before and in the 1830s, when clear‐cutting was strictly forbidden in BF (Genko, 1902–1903). Neither obvious growth releases suggesting harvesting of neighboring trees (Lorimer & Frelich, 1989; Montoro Girona, Rossi, Lussier, Walsh, & Morin, 2017), nor growth depressions associated with defoliation by insects (Langström, Annila, Hellqvist, Varama, & Niemelä, 2001) and compression wood reflecting wind disturbance (Zielonka, Holeksa, Fleischer, & Kapusta, 2010) were found in the cores synchronic with the establishment of those cohorts. Likewise, nothing similar was found in the latter cohort in the 1880s (data not shown). This indicates that intense clear‐cutting, insect outbreak and windthrow are very unlikely to be the main drivers of oak and pine population dynamics in the studied stand. Furthermore, a canopy‐opening disturbance alone would not remove the competition from other tree species in this mesic habitat type, which would likely strongly limit or even inhibit the establishment of shade‐intolerant oak and pine (cf. Aldrich et al., 2010). Additionally, both oak and pine seedlings were proven to benefit from the improvement in seedbed conditions resulting from burning (Brose, Dey, Phillips, & Waldrop, 2013; Brose et al., 2014; Hille & den Ouden, 2004).

The shift in fire regime which occurred in our study site in the first decades of the 1800s promoted spruce as well, compared to the period prior to the 19th century. Spruce encroachment is a strong indicator of relaxation in both fire intensity and frequency. Fires of sufficiently low intensity may not kill the seedlings, nor do they provoke mortality in the overstory. Thus, especially when combined with successively longer fire intervals, these shade‐tolerant tree species could have gained a strong competitive advantage in the overshadowing conditions of the stand (Niklasson, 1998; Niklasson et al., 2010; Zin, 2016) at the expense of oak and pine regeneration, which in turn ceased after the 1880s.

### 4.3 The mesophication era: establishment of the shade‐tolerant tree species after the 1920s

Since the beginning of the 20th century, tree recruitment was dominated by mesophytes such as *Carpinus*, *Tilia*, *Acer*. The regeneration of these species is an indicator for low light conditions in the stand (Ellenberg, 1996). Consistent with the results of studies from North America, we recorded exclusive regeneration of these species at the cost of species with intermediate or high light demands, in line with the described mesophication cycle (Nowacki & Abrams, 2008). The complete recession of fire events in this period is concurrent with the regeneration of fire‐sensitive species; the last abrupt regeneration pulse of spruce tracked well the last fire event, in 1920.

When compared to the two previous periods of tree succession, evidenced by our study, the mesophication era brought higher diversity among tree taxa—a phenomenon well documented across temperate North America (Flatley et al., 2015; Nowacki & Abrams, 2008). However, this may not mean a general increase in plant diversity of the site since the ground flora richness likely decreased in result of fire exclusion and the following increase in canopy closure (Brewer, Abbott, & Moyer, 2015; Deák et al., 2014). Still, the empirical data from temperate European oak ecosystems is limited and reports often contrasting effects of fire disturbance on groundcover plant abundance (Deák et al., 2014; Ziobro et al., 2016), so final concluding on the mesophication effect on vegetation diversity of our stand has to stay limited to the tree species level, which we studied.

We suggest that the situation of increased tree taxa diversity which we documented is likely to change in future in the absence of fire disturbance since (a) the light‐demanding old‐growth oak and pine populations will gradually die out with no effective regeneration due to closed‐canopy stand conditions and (b) the ongoing establishment of shade‐tolerant mesophytic broadleaves (cf. Figures 2 and 3) will further facilitate their dominance by decreasing fuel bed flammability.

Gradual reduction in flammability resulting from the mesophication has been ascribed mainly to the microclimate alteration caused by shading (in effect of increasing stand density and high leaf area) and to the specific features of leaf litter and woody debris of mesophytic tree taxa. Higher packing ratio and lower resistance to decay due to the lower lignin content of litter place fuel beds produced by these trees much below the flammability level of those produced by oak and pine species, in turn further facilitating the mesophication cycle (Nowacki & Abrams, 2008 and references therein). Even though there is still no data to confirm this for European conditions, we use broad evidence from North America to predict that previously dominating oak and pine communities may become eliminated by the expansion of mesophytic tree species in the absence of fire (Flatley et al., 2015; Lafon et al., 2017; Nowacki & Abrams, 2015).

Besides fire, mammalian herbivory has been proven to be of crucial importance in shaping tree regeneration and hence forest composition, both in BF (Churski et al., 2017; Kuijper, Cromsigt, et al., 2010; Kuijper, Jędrzejewska, et al., 2010b) and worldwide (Côté, Rooney, Tremblay, Dussault, & Waller, 2004; Forrester, Lorimer, Dyer, Gower, & Mladenoff, 2014). The possible interacting effect of those two main consumers of plant biomass (Bond & Keeley, 2005) should be considered as well (cf. Nowacki & Abrams, 2008). In our study site browsing was likely enhancing the shift from oak and pine to hornbeam and lime. Even though the palatability does not differ considerably among these species, oak and pine are more sensitive to browsing by ungulates than the latter species. Hornbeam and lime can tolerate high levels of browsing due to their adaptive traits (Churski et al., 2017; Kuijper, Cromsigt, et al., 2010). Despite this, our annually resolved record of tree regeneration and fire history suggests that the loss of fire was having an overriding effect since the onset of both lime and hornbeam regeneration occurred much later than the documented rise in ungulate numbers in BF, dating to the late 1800s (Jędrzejewska et al., 1997). In the present situation however, in a forest ecosystem devoid of fire, herbivory is likely strongly influencing the ongoing mesophication by shaping the relation between different shade‐tolerant, fire‐sensitive tree species with contrasting adaptations to browsing (cf. Churski et al., 2017).

## 5 CONCLUSION

To our knowledge, this study is the first in temperate Europe reconstructing and analyzing the long‐term compositional changes of a mixed deciduous forest and linking them to alterations of fire regime, with unequivocal record of fires from the studied stand itself. The recorded tree succession is consistent with data on well‐documented mesophication resulting from fire suppression across temperate forests of Eastern North America (Flatley et al., 2015; Lafon et al., 2017; Nowacki & Abrams, 2008). In that biome, altered disturbance regimes (and specifically fire exclusion) were proven to be more important than climate in driving long‐term vegetation change (Nowacki & Abrams, 2015), thus challenging both the classic forest ecology models which assume climate and resources, mainly soil conditions, to be main determinants of forest composition (Oliver & Larson, 1996; White & Pickett, 1985), as well as much of the ongoing debate on climate warming effects on future plant communities (e.g., Hansen et al., 2001; Kirschbaum, 2000). However, one must acknowledge that climate influence on forest succession is complex and not always possible to be completely disentangled from other relevant factors, including fire disturbance (e.g., Bhatta & Vetaas, 2016; Pederson et al., 2015).

The documented compositional changes are similar in our study site to the core area of the Polish Białowieża National Park which is under no management since 1921. In that area, an evident decline in both oak and pine establishment has also been recorded over the last decades (Brzeziecki et al., 2016; Kuijper, Jędrzejewska, et al., 2010b), with not a single successful pine recruit in the last 76 years (Brzeziecki et al., 2016). Since most of the BF area was subjected to frequent fires from at least the mid‐1600s until the mid‐1800s and later (Brincken, 1826; Niklasson et al., 2010; Zin et al., 2015), we propose that the recession of pine in BF is better explained by its response to changes in the fire regime rather (Niklasson et al., 2010; Zin et al., 2015) than to changes in the forest management and/or herbivory pressure (Brzeziecki et al., 2016). Our study shows that an open environment due to frequent burning and less competition from fire‐sensitive species were likely the prerequisites not only for pine, but also for oak dominance in BF.

We argue that the widespread idea that European temperate forests are nonflammable ecosystems (Ellenberg, 1996; Vera, 2000) should be questioned. Fire impact in mesic, deciduous‐dominated forest habitats might not be as straightforward to understand as in xeric, coniferous habitats (Niklasson et al., 2010; Zin et al., 2015). Due to the general lack of documentation, the knowledge of local fire regimes (fuel dynamics, flammability, spread rates, intensities, effects on tree species, etc.) in this forest type is practically nonexistent in Europe, which underlines the need to continue studying forest succession in relation to the role of fire and other factors in that region. This is also important for predicting forest development under imminent and anticipated changes in climate, as well as to incorporate it into nature conservation and management practices, especially in highly valuable nature areas like Białowieża Forest.

## CONFLICT OF INTEREST

None declared.

## AUTHOR CONTRIBUTIONS

MN and EZ conceived the ideas and designed methodology and study; AS and EZ collected the data, carried out and completed the laboratory work, performed literature review and data analysis; AS lead the writing of the manuscript with significant input from EZ and MN. All authors contributed critically to the drafts and gave final approval for publication.

## Data Availability

Fire history and tree succession reconstruction from a 43‐ha mixed deciduous stand in Białowieża Forest: https://doi.org/10.5061/dryad.3ffbg79dj.
