# Supplementary material for: Mesophication in temperate Europe: A dendrochronological reconstruction of tree succession and fires in a mixed deciduous stand in Białowieża Forest
Source: Ecol Evol. 2020 Jan 10;10(2):1029–41. doi: 10.1002/ece3.5966 (PMC6988544; doi:10.1002/ece3.5966)
Supplement: Supplementary file 1 [file ECE3-10-1029-s001.docx]

**APPENDICES:**

**APPENDIX 1.** Enhanced cell contrast with zinc paste and water for age determination of two tree seedlings (*Carpinus betulus* L., *Quercus robur* L.). [6–40 × magnification].


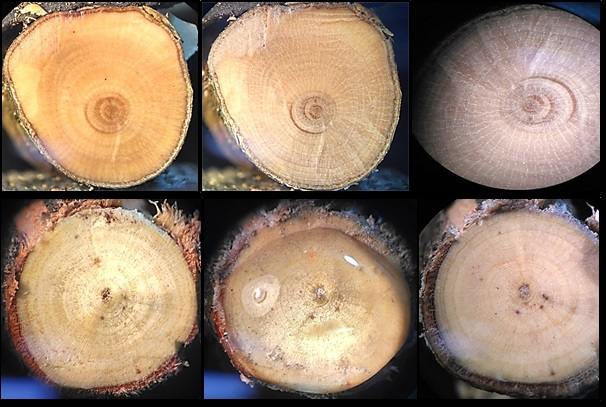


Appendix 1

**APPENDIX 2.** The regression equations (height x age) for the local growth model for *Quercus robur* L., *Pinus sylvestris* L., *Carpinus betulus* L., and *Picea abies* L. Karst.

*Q. robur*: ~ 9 yrs to reach 20 cm, followed by 16 cm/yr. Derived from 17 core pairs;

*P. sylvestris*: ~7 yrs to reach 25 cm with a following growth of 27 cm/yr. Derived from 12 core pairs;

*C. betulus*: A simple linear regression model was used to predict age based on height:

$LnAge\times1.58+0.04\times Height$, (R^2^= 0.123). Derived from 42 seedlings;

*Picea abies* L. Karst. samples illustrated inconsistent early growth (min.-max.: 4 cm/yr – 21.4 cm/yr). Investigated from 13 core pairs; 2 seedlings

*
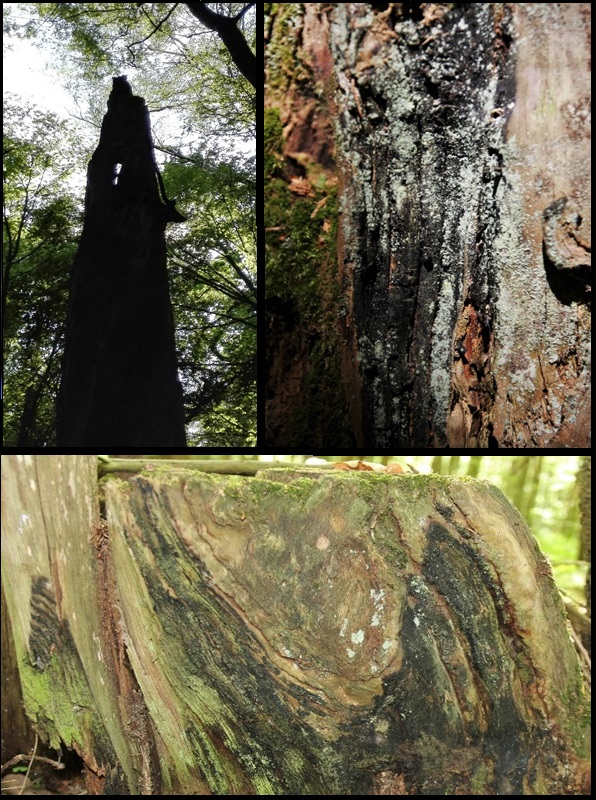
*

Appendix 3

**Appendix 3.** Fire evidence from the study site: a multiple-scarred Scots pine (*Pinus sylvestris* L.) stump in the field.


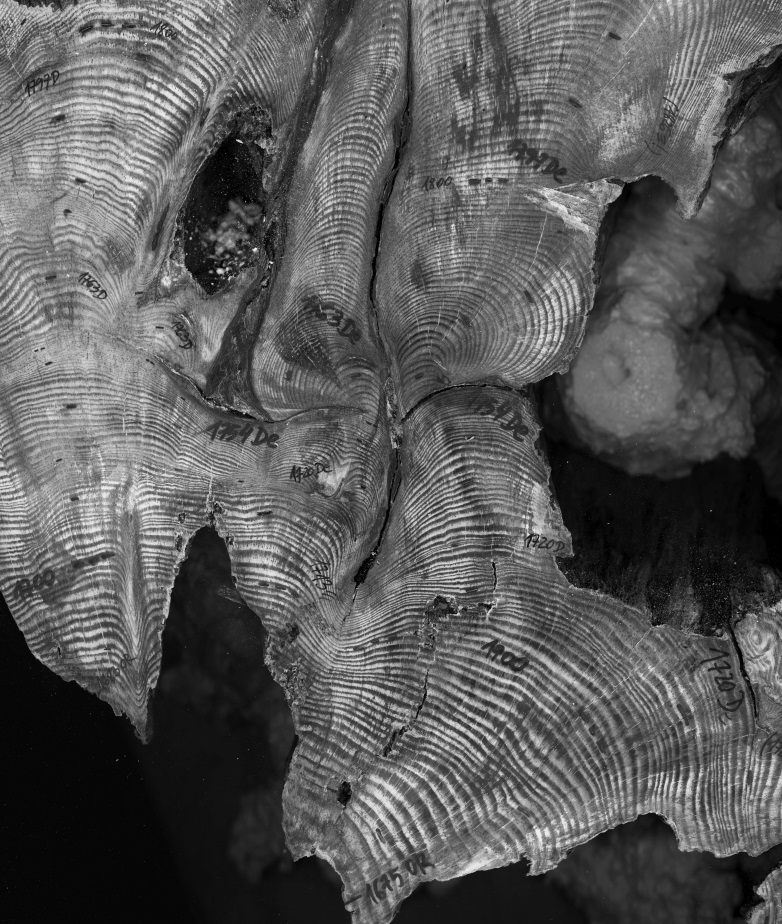


Appendix 4

**Appendix 4.** A cross-section from *Pinus sylvestris* L. stump with visible fire scars (FS) in: 1720, 1754, 1763, 1797. Tree ring span of this sample: 1675–1955 (youngest rings are not included in the picture).
